# Supplementary material for: Chromothripsis during telomere crisis is independent of NHEJ, and consistent with a replicative origin
Source: Genome Res. 2019 May;29(5):737–49. doi: 10.1101/gr.240705.118 (PMC6499312; doi:10.1101/gr.240705.118)
Supplement: Supplemental Material [file supp_gr.240705.118_Supplemental_file_1.zip › contigs/annotated_contigs/DB78/contig.2.DB78_length_493_mean_cov_11.8661257606.docx]

**DB78_length_493_mean_cov_11.8661257606**

```
ATCTCCGTCCACTGGCCCAGCCTGGCTCCCTGCCACCGCCACTGCCTCTGCCTCCACACTTCAGAAGAAAGACTTTACAGGAGCCGCAG
 >chr7:131530653-131530833 - E=1e-96
ATGATGCTGAACTTCAATTAGGCCCTGCTGGCAAATTAGTCACGCAGGGTGACATTTTCCAATCATAGAACATTAGCATCCCCCCTAAT

TA|GCA|TCCTGATCAAGCCAGAAGCTCACCATTGTTCCTGCGCAGCTAAGTGCCCAGGTTCATCCTAATTGAGCTGAACACTAGTTGC
      >chr7:131505564-131505874 - E=5e-172
TGGGTTCCATGGTTCTCTTCTATGACCCACAGCTTCTAATAGAGCTATAACACTCACCCCATGGCCCAAGGTTCCATTCCTTGGAATCC

GTGAGGCCAAGAACCCCAGGTCGGACAACAAAAGGCCTTCTGCCATCTTAGGAGCGGCCACCACCATCTTGGGAGCTCTAAGAACAAAG

ACCATCTGGTAACATTATGACCTGAGTTGGGGTAGTGGTTAATGGCCTTA
```
